# Suture Tendons

**Published:** 1854-12

**Authors:** M. Chassaignac


*Suture of Tendons.* By M. Chassaignac.

Our readers may recollect a case of successful tenoraphy practiced by Professor Sedillot of Strasbourg, which was reported in a late number of this journal. In the Compte Rendu de la Societe de Chirurgie de Paris, for April, 1854, we find a second case in which this operation was successfully performed, and which appoars to us worthy of being brought to thc notice of the profession.

It relates to a girl of sixteen years, who, in Nov , 1853, fell on a piece of broken glass, and received a transverse wound on the anterior aspect of the lower part of the fore arm, which, after süppurating for a time, cicatrized.

Some months subsequently the patient entered the Hospital St. Antoine with the inability to flex the index finger. It was found that the inferior end of the tendon which had been divided was attached to the cicatrix of the wound already described, and that when this cicatrix was moved, the index finger moved also. On the fourth of February, M. Chassaignac laid bare the flexor tendons, by dissecting a rectangular flap of integument from the fore-arm, and passed a suture through the divided portions of the flexor of the index finger. The flap was then replaced and secured by stitches, and the hand was strongly flexed. In a fortnight the wound had united, and the patient left the hospital with the perfect use of her finger. The ends of the tendon were not refreshed. This fact, when considered in connection with Dr. Mayo's case of rupture of the ligament of the patella, and our observations on tendinous ruptures in the first number of the present volume of this journal, leads to the conclusion that exact apposition is all that is necessary for the re-union of divided tendons, even when separation has existed for a comparatively long period.

